# A Pilot Investigation of the Relationship between Climate Variability and Milk Compounds under the Bootstrap Technique

**DOI:** 10.3390/foods4030420

**Published:** 2015-09-11

**Authors:** Mohammad Reza Marami Milani, Andreas Hense, Elham Rahmani, Angelika Ploeger

**Affiliations:** 1 Department of Organic Food Quality and Food Culture, University of Kassel, Nordbahnhofstr. 1a, 37213 Witzenhausen, Germany; E-Mail: a.ploeger@uni-kassel.de; 2 Meteorological Institute, University of Bonn, Auf dem Hügel 20, 53121 Bonn, Germany; E-Mails: ahense@uni-bonn.de (A.H.); erahmani@uni-bonn.de (E.R.)

**Keywords:** climate variability, THI, ETI, milk compounds, bootstrap, uncertainty, Iran

## Abstract

This study analyzes the linear relationship between climate variables and milk components in Iran by applying bootstrapping to include and assess the uncertainty. The climate parameters, Temperature Humidity Index (THI) and Equivalent Temperature Index (ETI) are computed from the NASA-Modern Era Retrospective-Analysis for Research and Applications (NASA-MERRA) reanalysis (2002–2010). Milk data for fat, protein (measured on fresh matter bases), and milk yield are taken from 936,227 milk records for the same period, using cows fed by natural pasture from April to September. Confidence intervals for the regression model are calculated using the bootstrap technique. This method is applied to the original times series, generating statistically equivalent surrogate samples. As a result, despite the short time data and the related uncertainties, an interesting behavior of the relationships between milk compound and the climate parameters is visible. During spring only, a weak dependency of milk yield and climate variations is obvious, while fat and protein concentrations show reasonable correlations. In summer, milk yield shows a similar level of relationship with ETI, but not with temperature and THI. We suggest this methodology for studies in the field of the impacts of climate change and agriculture, also environment and food with short-term data.

## 1. Introduction

It is well documented that agriculture production, farm economy, milk yield and milk components and agricultural livestock welfare are impacted by climate variability [1].

The general increase of human population and the amount of dairy products in human diet requires on average a two percent growth of global milk production to be able to amend the increasing demand for dairy products. Furthermore, there is no doubt that cows should live in an optimum environmental condition (animal welfare aspects) to be productive in both quantitative and qualitative aspects [2].

However, the definitions of optimal environmental conditions, as well as the sensitivity of milk productivity by deviations from that optimal range, are still not clear. This holds especially true for developing countries and emerging economies. Therefore, the effects of climate variability upon milk yield, as well as amount of milk fat and milk protein (g/100 mL fresh milk), are investigated with data from Iran.

The aim of this research is investigating the effect of climate variability on three main components of milk.

According to the report of United Nation in 2013, the population in Iran will increase about 43% by 2050 up to 115 million [3]. Consumption of milk per person in Iran is 30 to 150 kg/capita/year as reported by Food and Agriculture Organization. Due to increasing human population and urbanization, the demand for food and animal production are expected to be enhanced. It shows the importance of the interdisciplinary research related to the effects of climate change on food security. Unfortunately such research has been done less frequently in the Middle East and Iran as well.

Previous and ongoing research [1,4,5,6,7,8,9] determined that tolerance of climate variability in ecosystems plays important role on fodder, rate of ability of cow physiological adaptation, ruminal fermentation, cow nutrition, husbandry systems, and has an effect on DNA integrity. The quality and quantity of milk compounds is generally a result of complex interactions of variables. These are not fixed and they can change with the time of the year, environmental conditions, and climate variability. The yield of Holstein cattle is found to be more sensitive to climate than those of Jersey cattle, but Jersey milk composition is more sensitive to climatic influences. The effect of heat stress on decreasing milk yield is more highlighted in Holstein rather than in Jersey cattle [10].

Already, three decades ago, Rodriquez [11] investigated the effect of temperature on milk composition and yield in Florida by the means of an analysis of variance for cows of Holstein and Jersey breed with monthly averages of single-day milk samples. They reported that yield increases if the maximum daily temperature increases from 8 to 29 °C. However, they also report a rapid decrease when maximum daily temperatures are higher than 29 °C. In contrast, fat and protein decline over the entire range, from 8 to 37 °C.

For including humidity and temperature as physiological derive of milk productivity index, combinations such as the temperature humidity index (THI) have been suggested as an indicator for thermal stress [12,13,14].

However, THI does not consider the loss of energy by passive cooling, e.g., through turbulent motion in the atmosphere. This is commonly parameterized by wind speed. Therefore, the equivalent temperature index (ETI) is used in this investigation as another climatic index, which incorporates wind speed, temperature, and humidity.

The combination of milk productivity data (yield, fat and protein concentration in milk) with physiologically relevant meteorological data and indices, taken from a third generation atmospheric reanalysis for Iran and the Middle East, is unique.

The major question to be answered is whether, indeed, large scale patterns of meteorological parameters can be identified that provide a statistically significant and physiological relevant influence upon milk productivity in terms of yield and relevant nutrient content in the milk.

The rather short record availability (2002–2010) for climate variability purpose requires that special statistical methods are applied to avoid misinterpretations due to over-fitting. Milk component data in Iran is provided for fat, protein (g/100 mL fresh milk), and milk yield, in spring and summer, from the Animal Breeding Centre of Iran. Climate data, including daily averages of temperature in two meter height (T_2m_), wind speed, sea level pressure, and specific humidity, were taken from the Modern Era Retrospective-Analysis for Research and Applications (MERRA), which is undertaken by NASA’s global modeling and assimilation office [15].

This paper focuses on data preparation and on introducing some statistical methods to cope with uncertainties in special data sets. Further climate indices are selected from the literature, which aggregate more climatic parameters in a physiologically meaningful way. Finally the paper is looking, as a pilot study, for some basic relations between dependent and independent variables.

For investigating the statistical relation between the variables in this multidimensional context, in a first step, simple linear regression is chosen because it is easier to use and to interpret. The particular choice of linear regression and correlation analysis is motivated by future investigations on the multivariate regression relations between climate variables and milk productions.

## 2. Materials and Methods

### 2.1. General Information on Study Domain

Iran is placed in Southwestern Asia, approximately at latitudes 25°00′ N–38°39′ N and longitudes 44°00′ E–63°25′ E, and covers about 1,648,000 square kilometers with a population of more than 77 million.

Topography in Iran plays a very important role on climate conditions. It is bounded on the north by the Caspian Sea, and, on south, by the Persian Gulf and the Gulf of Oman. There are two main mountain chains in Iran, named the Alborz and the Zagros, which dominate Iran in the north and northwest-southeast, respectively. These mountains have a significant effect on distributing perceptible atmospheric water vapor. Therefore, the central and eastern parts of Iran receive less precipitation than the north and west [16,17].

According to Ahrens [18], two thirds of Iran has, mostly, two climate categories: arid and semi-arid, based on the Köppen climate classification. Previously, eight climatic zones have been categorized in Iran according to a cluster analysis of rainfall [19].

**Figure 1:**
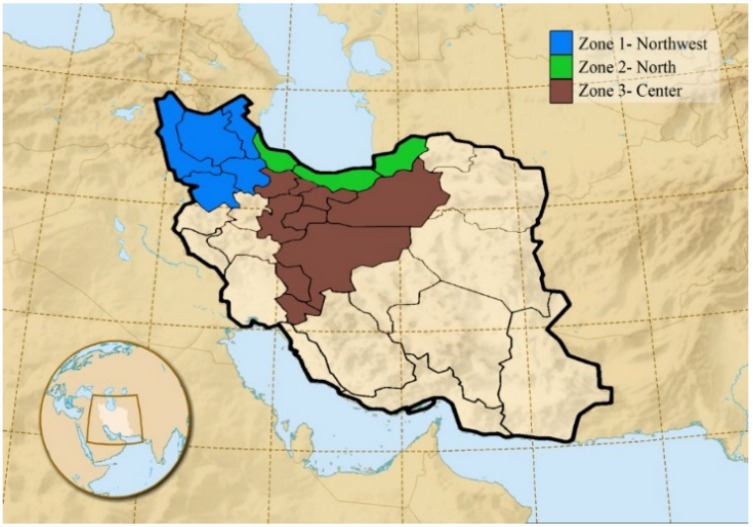
Classification of three study zones according to climate conditions in Iran [19,20].

In this study, our study area consisted of three zones, according to different climate conditions and data availability (Figure 1). The first zone is in Northwest Iran, with cold and semi-arid climate conditions. The second zone is placed in the north, with a Caspian, mild and humid climate. The third zone is almost in the center of Iran with arid and semi-arid, mild and semi-warm climate conditions [20].

In Iran there are 25,353 industrial herd stations with a capacity of about 3.3 million cows. At the moment, 18,295 industrial herd stations with about 1.3 million cows are active. Sixty-six percent of cows in Iran have a pure genetic origin (Holstein, Jersey and Brown-Swiss), 27% are hybrid and 7% are home born. About 64% of the pure cows are Holstein and 2% are Jersey and Brown-Swiss. Additionally, 19.3% of the hybrid cows have at least one Holstein parent. The total milk yield in 2013 was 3792 thousand tons. Most feed ingredients include: moisture corn, grain, alfalfa, straw, silage, wheat, soybean, cottonseed meal, and other forage [21].

### 2.2. Milk Data Conditions

Gathering milk data needs attention to some conditions. The most important factors in milk composition, which should be considered, are genetic, environmental conditions, management of feeding, season, cow age, rating of lactation, and pregnancy; these conditions provide a preprocessing of the milk data set of the investigation.

The yield of milk reduces when the non-lactating (dry) period is less than 40 to 45 days for first lactation and 55 to 65 days after second [22]. Milk yield also increases with increasing body weight, age, and number of lactations [23]. During the first to fifth lactation, milk fat decreases each year by about 0.2 (g/100 mL milk) and protein drops by 0.02 (g/100 mL milk) to 0.05 (g/100 mL milk) per year [24].

Mastitis, which is one of the most important and prevalent illnesses in cows, has the greatest effect on milk yield [25].

In this study, some main compounds of Holstein cow’s milk in Iran were selected. Milk data is collected from 2002 to 2010, from almost 600 industrial herd stations, with about 1.2 million milk records of Holsteins cows from the seasons in which cows use natural pastures or fresh fodder. This is to say, during the months of April to September.

Industrial herd stations have been chosen because they consider the technical and health control management system, milking twice a day and a controlled nutritional feeding. They also have a regular data recording system, which was a very important aspect for this study.

All these conditions are taken into account when calculating from the raw data estimated averages of milk yield, fat, and protein concentrations of fresh milk. The milk data of the cows between their third and sixth calves were considered and the data of cows with the problem of mastitis are not considered. Additionally, we chose industrial herd stations with good health services, under controlled conditions and with veterinary care.

For the milk parameters, the yield of milk (kg/Year), fat content (g/100 mL milk), and protein content (g/100 mL milk), were used, from which the values are monthly averages in all herds and stations, respectively, in each zone. Then, the milk data bank is fixed under the mentioned conditions (lactation, health, *etc.*) with 936,227 milk records of Holstein cows from 2002 to 2010 in spring and summer.

The milk productivity data are directly calculated from the available data after correction for sampling and physiology, as described above.

### 2.3. Climate Data Basis and Climate Parameters

The climate database was set up for the same period as for the milk productivity data, and covers the area of Iran. As specific parameters, the daily averages of T_2m_, sea level pressure, specific humidity, and the wind component vectors from MERRA reanalysis with a resolution of 1/2 degrees latitude and 2/3 degrees longitude are chosen. Thus, for each zone, the area, through the latitude and longitude range, and averaged the climatic parameter for all grid points in the adaptive zone, was considered. The other climate parameters, such as vapor pressure (*e*), saturation vapor pressure ($e_{w}^{*}$), relative humidity (*RH*), equivalent temperature index (*ETI*), and temperature humidity index (*THI*) were calculated by the following expressions.

(1) e=1.6077×p×q

where, *e* is the water vapor pressure (hPa), *p* is the sea level pressure (hPa), and *q* is the specific humidity.

(2) $$e_{w}^{*}=6.1078\exp[(17.1\times T)/(235+T)]$$

where $e_{w}^{*}$ is saturation vapor pressure (hPa), which is calculated using the Magnus Equation from the two meter temperature, *T* (°C) [26].

Relative humidity (%) is calculated as:

(3) $$RH=e/e_{w}^{*}$$

To assess the risk of heat stress, which is an important aspect in livestock health in humid and hot climates, we applied *THI*, which is a function of air temperature in two meter height, *T* and dew point, *T_d_*.

Dew point is the temperature at which the actual water vapor pressure equals the saturation water vapor pressure [14,27].

*T_d_* can be calculated by the equation below, or from the Magnus Equation, by using the saturation vapor pressure.

(4) $$T_{d}={\left(\frac{RH}{4}\right)}^{\frac{1}{8}}\times [112+(\frac{9T}{40})]+\frac{T}{40}-112$$

(5) $$THI=41.5+T+0.36T_{d}$$

where $T_{d}$ is the dew point temperature, T is temperature in two meter height and *RH* is relative humidity (%).

Additionally, we calculated the *ETI*, which takes into account the loss of energy from the animal’s body by conduction and turbulent transfer [28,29,30]. The *ETI* is calculated as follows [31]:

(6) $$\begin{array}{l} ETI=27.88-0.456T+0.010754T^{2}-0.4905RH+0.00088RH^{2}+\\ 1.1507V-0.12645V^{2}+0.019876T\times RH-0.046313T\times V \end{array}$$

where *V* is wind speed (m/s) derived for the zonal and meridional wind components. Note that the nonlinear relations between dew point temperature, water vapor pressure, and specific humidity in *THI* and between temperature, relative humidity, and wind velocity in *ETI* require daily input data to evaluate monthly means.

### 2.4. Bootstrap Technique

Temporally short records of data and their statistical analyses, as in the present case, require careful considerations of their inherent uncertainties. This is necessary in order to not to come up with misleading conclusions due to the small sample size. This investigation is based on a comparable large dataset of milk compound data from the Holstein breed and from various regions in Iran. However, if the intention is to find statistically meaningful connections to the inter-annual variability of near surface climate, the sample size decreases dramatically because only nine years of data (2002–2010) are available to estimate the dependency between climate variations and milk compound changes. Although we consider in this paper the results of a nine year period of climate conditions on milk compounds and yield with a high number of cows but for climate research such a period is relatively short. Considering monthly values is not helpful due to the ever-present annual cycle, which makes, for example, direct comparisons of actual spring and summer values practically meaningless (“summer is always warmer than spring”).

Bootstrap techniques are a very valuable, computer-based tool to assess the influence of uncertainty within the available data sample on the results of the statistical analysis [32].

Bootstrapping belongs to a class of resampling techniques. Based on the empirical probability distribution function, or cumulative distribution function (cdf), of the data sample, new realizations of the sample are generated, which share, by this construction, the same distribution function. Depending on the way in which the cdf is estimated from the original sample, one distinguishes between parametric and non-parametric bootstrapping. In the first case, parameters of an assumed cdf are estimated from the original sample (e.g., mean and variance for a Gaussian cdf). Plugging these parameter values into the cdf allows one to generate, by drawing at random from this fully defined cdf, new sample values [33]. The second way to generate the new bootstrap samples is by estimating the cdf non-parametrically through sorting of the original sample in increasing order. Sampling at random from this empirical cdf leads directly to a random sampling of the original data set with replacements such that, in the new sample, some of the original data are left out and replaced by copies of the remaining original data.

**Figure 2:**
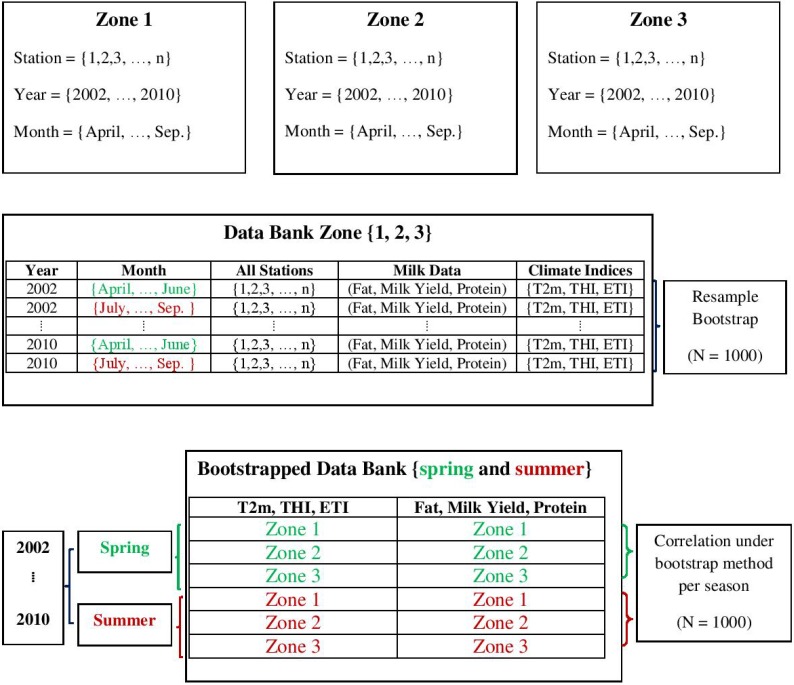
Flowchart of data set.

In the present case, the non-parametric bootstrap technique was applied in two different situations. As mentioned above, the milk compound data set is quite comprehensive if taken by itself. However, comparing it to climate data requires a reduction of these data to monthly averages in order to concentrate the analysis on the relevant time scales of months to years. Therefore, in a first step, we applied the bootstrap technique to data from each station (consisting of herds) in each zone, separately generating 1000 samples of monthly means of milk yield, fat, and protein concentrations for the full period of 2002–2010, which are presented in Figure 2.

The general aim is to compare the results from the bootstrapped correlation analysis with those from the classical approach in order to assess the robustness of the linear correlation coefficients under the resampling of this rather small data set in terms of climate variability.

Technically, we implemented the analysis using R version 3.0.2, which provides various packages for statistical data analyzing, calculating, and graphical display [34].

### 2.5. Linear Relationships between Climatic Parameters and Milk Compounds

The linear relations between the climatic parameters or indexes (T_2m_, THI, ETI) and the milk compounds (milk yield, protein, fat) have been estimated through correlation analysis under bootstrapping. The flowchart of the used data is shown in Figure 2.

Correlation in time series data, as in our case, can be controversial. If there is no obvious relationship between parameters, it could be very useful and relative easy to apply.

Correlation between variables x and y with Gaussian distributions and variances $\sigma_{x}^{2}$ and $\sigma_{y}^{2}$, respectively, with the range of [−1, +1] is defined by:

(7) $$\rho_{\text{xy}}=\frac{COV(x,y)}{\sigma_{x}\sigma_{y}}\;$$

We applied bootstrap with *N* = 1000 resampling steps. Then, we have 1000 samples of x  and  y as $x=\;\left(x_{1},x_{2},\ldots ,x_{N}\right)$, and $y=\;\left(y_{1},y_{2},\ldots ,,\ldots ,y_{N}\right)$. Correlation of the transformed variables of x and y is estimated as:

(8) $$r_{\text{xy}}=\frac{\sum_{i=1}^{N}\left(x_{i}-\bar{x})(y_{i}-\bar{y}\right)}{{\left\{\sum_{i=1}^{N}{\left(x_{i}-\bar{x}\right)}^{2}\right\}}^{0.5}{\left\{\sum_{i=1}^{N}{\left(y_{i}-\bar{y}\right)}^{2}\right\}}^{0.5}}\;$$

We assessed the inherent uncertainties of bootstrapping as described above. Two separate seasons have been analyzed to account for the seasonal dependencies of the climatic input parameters and the physiological responses of cows during spring and summer. Additionally we distinguish between the relationships between the climate data and milk data, and those describing the inter-relationship between both data sources. The correlation analysis during the time period of 2002–2010 in spring and summer are presented in Section 3.2. In these matrices, the respective correlation coefficients are averaged from the correlations across all stations within each bootstrap sample, with sample size of 1000. We also calculated the confidence interval from inter-quartile range of the correlation coefficients across all bootstrap samples. Rahmani *et al.* [35] also applied a bootstrapping technique for estimating the sampling uncertainty of the correlation and regression analyses.

## 3. Results and Discussion

### 3.1. Statistics of Climatic and Milk Parameters

**Figure 3:**
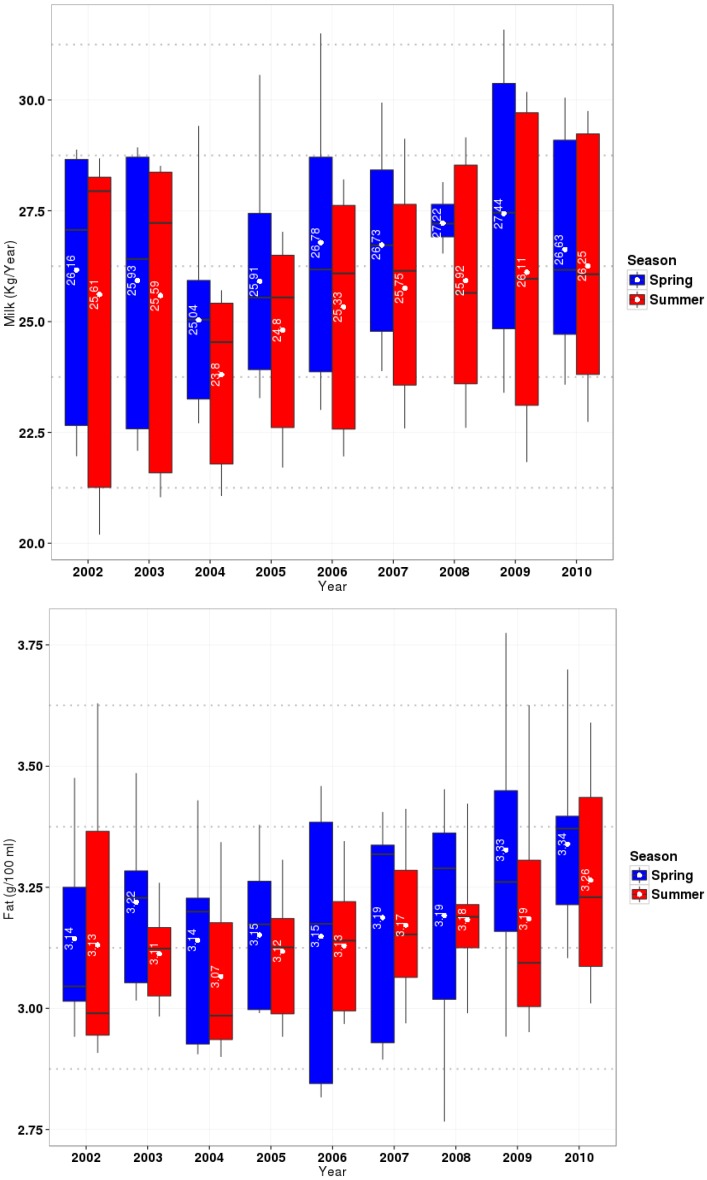

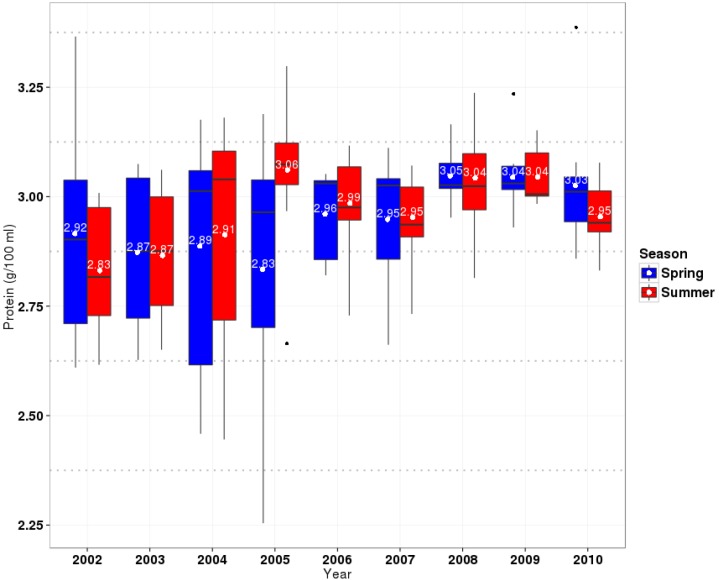
Statistics of milk parameters. Lines inside the box plots show the median and points show the season averages of milk compounds (Milk yield (Kg/Year), fat (g/100 mL), protein (g/100 mL) in fresh product) in spring (blue boxes) and summer (red boxes) from 2002 to 2010.

**Figure 4:**
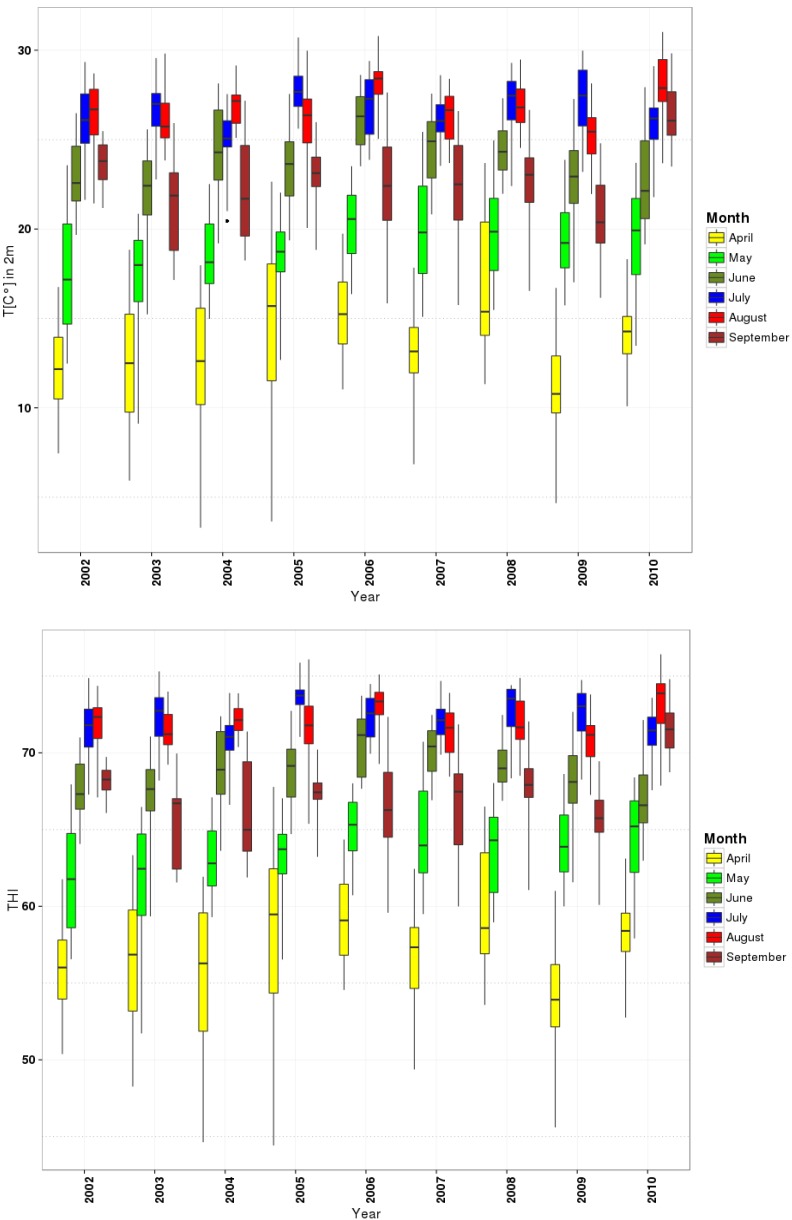

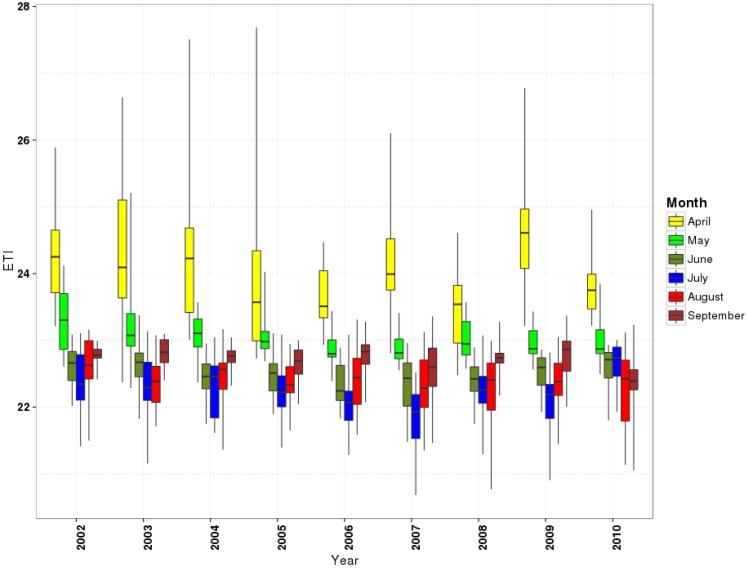
Statistics of climatic parameters from the box plot of T_2m_ (°C), THI, ETI, from 2002 to 2010 in spring and summer.

To obtain an overview of the relevant conditions for the relationship between milk productivity (amount and nutrient content) and climate variability, the statistics of milk parameters in Figure 3 and the climatic parameters in Table 1 and Figure 4 are summarized for short-term data (2002–2010) in summer (July, August, and September) and spring (April, May, and June), separately, in the study area.

**Table 1:** Temperature in two meter height (T_2m_ in °C), Temperature Humidity Index (THI) and Equivalent Temperature Index (ETI) and number of days that cow is under stress (NDS), during 2002 to 2010 in spring and summer.

	Spring			Summer		
	**T_2m_**	**THI**	**ETI**	**T_2m_**	**THI**	**ETI**
Minimum	8.0	50.7	22.0	17.1	61.83	21.21
Median	19.7	64.3	22.9	25.0	70.41	22.50
Average	18.7	63.1	23.2	25.3	70.43	22.44
Maximum	28.6	73.1	25.6	31.0	75.20	23.18
NDS	31	6	-----	168	100	-----

For milk parameters, yield of milk (kg/Year), fat, and protein (g/100 mL milk) are considered.

The box plots of milk compounds represent the scales of variability across all stations and years. There is hardly any visible seasonal signal in the three compounds of milk, which justifies using only the two seasons, spring and summer.

The results of Figure 3 indicate that from 2002 to 2010, the monthly average of milk yield decreased from spring to summer. The average of protein from spring to summer showed different trends, while the average of fat values decreased in an unremarkable way during this period. The maximum reducing rate of fat is for 2009, with 0.15 (g/100) mL milk (Figure 3).

Decreasing of milk yield is also reported by Rodriquez *et al.* and Bouraoui *et al.* [11,36]. They reported a decrease of protein and of milk yield. In this study, for protein is not big decreasing detected, which might be caused by differences in the experimental conditions.

As with the results in this investigation, Knapp and Grummer [37] and Roman-Ponce *et al.* [38] also did not find any significant relation between fat reductions and heat stress. Reduction in amounts of fat and protein from spring to summer is also reported by Bouraoui *et al.* [36].

The climate data are the directly available MERRA data (T_2m_) or the derived compounds (THI, ETI) calculated from the respective MERRA data sets. As additional information, the average number of days of stress for cows is given.

For climate parameters, the monthly average of daily T_2m_, minimum and maximum, average of calculated THI and ETI and their maximum and minimum values are presented in Table 1and Figure 4.

THI is determined to assess the risk of heat stress with critical values of 72, 78 and 82. When the THI rises above 72, cows are probably under heat stress. Values higher than 78 will seriously affect milk production. When THI exceeds 82, very severe heat stress on cow occurs with significant decreases in milk production [36,39].

Hence, 72 as the start point of heat stress was assumed and NDS presents the number of days with a THI above 72 in Table 1. For ETI, no critical value could be found from the literature that could serve as start point for stress. For temperature, the number of days exceeding 25 °C was considered. It is the upper critical temperature for Holstein cows that they are under stress (NDS) [40].

The monthly average values for T_2m_, THI and ETI, respectively, change from 18.7 °C, 63.1 and 23.2 in spring to 25.3 °C, 70.43 and 22.44 in summer.

The annual cycle of the climate parameters across all stations and their short-term changes, between 2002 and 2010, can be seen in Figure 4. Here, a very prominent annual cycle is apparent for all three parameters with a strong change during spring and a plateau during the summer months. Again this special configuration is the second justification for selecting the two seasons.

### 3.2. Results for Correlation between Climatic Parameters and Milk Compounds

**Figure 5:**
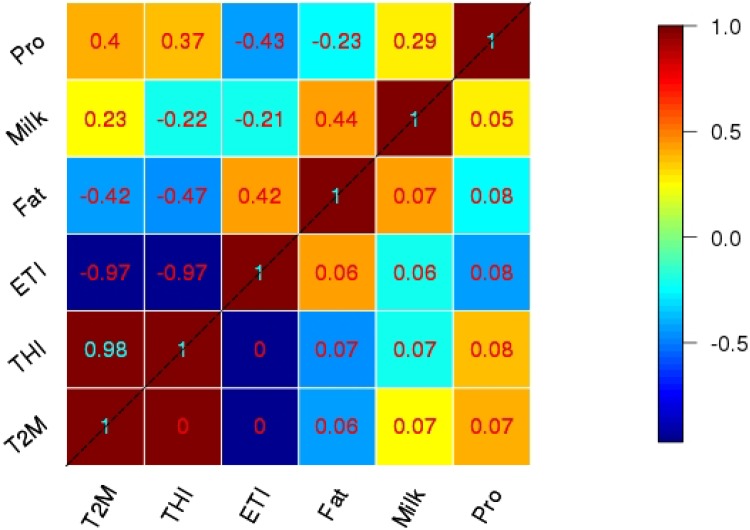
Correlation (above diagonal) and respective interquartile range of bootstrap sample (below diagonal) between milk compounds and climatic indices in spring.

**Figure 6:**
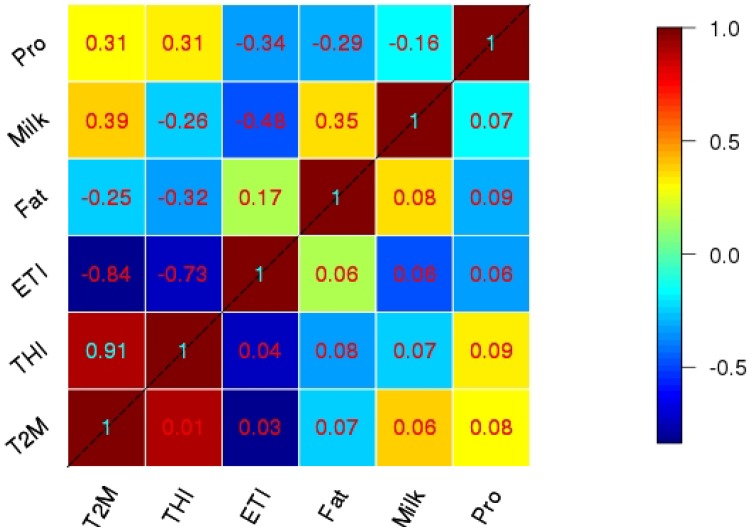
Correlation (above diagonal) and respective interquartile range of bootstrap sample (below diagonal) between milk compounds and climatic indices in summer.

Figure 5 and Figure 6 describe summarized results of the correlation analysis between T_2m_, THI, ETI and milk yield, fat, protein, in spring and summer, respectively.

The entries above the diagonal in these two matrices are the respective correlation coefficients as averages of the correlations across all stations during the time period of 2002–2010 within each bootstrap sample. Bootstrapping with a sample size of 1000 is applied to the total sample of size 81 (3 × 27), formed by all nine years at all three climatic zones (Figure 1). The values below the diagonal are estimated confidence intervals from the inter-quartile range of the correlation coefficients across all bootstrap samples. Inter-quartile range is the distance between 25% and 75% quantiles of the bootstrap samples.

The correlation results for spring (upper triangle in Figure 5) illustrate a positive correlation between milk yield with protein and fat content in the milk in spring, with the values of 0.29 and 0.44. This means that by increasing the milk yield, the fat and protein concentrations also increase. The correlations are weak and they explain, at most, 19% of the total variability (yield *vs.* fat with a correlation of 0.44). Correlations involving protein concentrations are also small compared to the sampling uncertainty (lower triangle in Figure 5).

In summer, milk yield still has positive correlation with fat (0.35), but, again, the correlation involving protein is small, showing that fat and protein concentrations seem to be independent of milk yield. This might be due to the rather short sample size, even when taking the different zones into account.

For both seasons, T_2m_ and THI show very high positive correlations with each other, and a high negative correlation with ETI, especially for spring. This means that, in contrast to the milk productivity data, we observe a very high dependency between the climatic variables, meaning that either variable can be chosen for analyzing the interdependency between climate variability and milk productivity. During summer, the correlations are still quite high, but the ETI has some independent information compared to T_2m_ and THI because the correlations drop to 0.7, meaning that only 50% of the total variance is common between ETI on the one hand and T_2m_/THI on the other.

Despite the small sample size for climate investigations (nine years but a very large number of milk parameters) and the related uncertainties, an interesting behavior of the relationships between milk compounds and the climate parameters is visible. During spring only, a very weak dependency of milk yield and climate variations is obvious, while fat and protein concentrations show reasonable correlations with an explained variance of around 15% to 22%. However, for summer, milk yield shows a similar level of relationship with T_2m_ and ETI, but not with THI. Also in summer the relationships of fat concentrations with the climate parameters level off, quite in contrast to the spring results.

**Figure 7:**
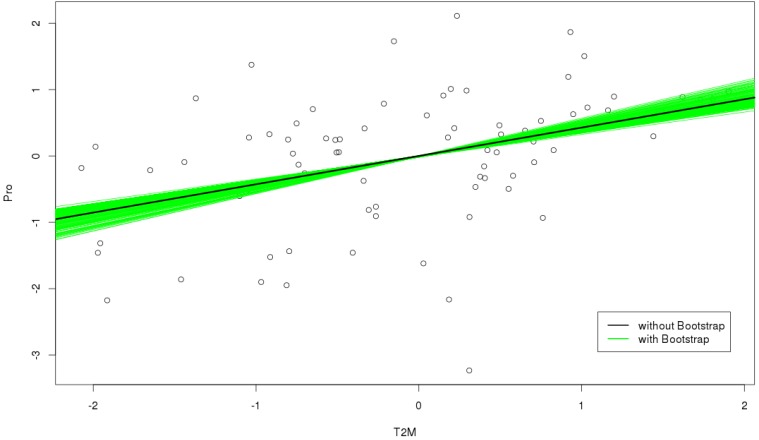

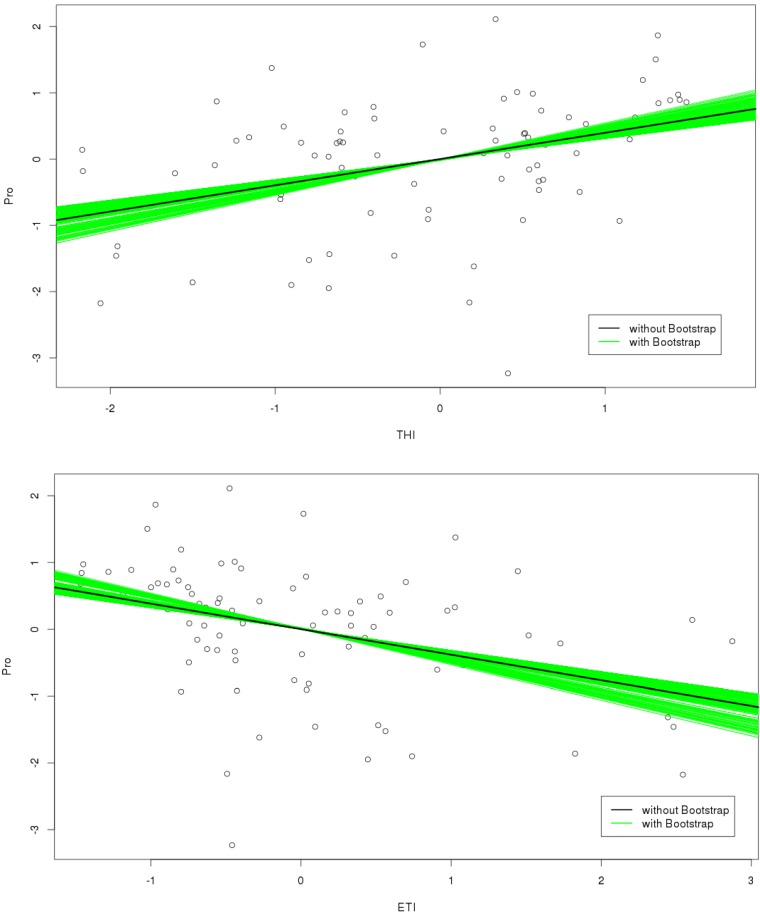
The best-fitted regression line before (black line) and after 1000 bootstrapping (green lines) between protein and T2M, THI and ETI indexes in spring.

Figure 7 presents some additional explanations regarding regression analysis between protein concentration, as predictand, and T2M, THI, ETI as predictors in spring, before and after applying bootstrapping. The data have been normalized to zero and a standard deviation of one. To that aim, we used anomalies of the data and applied the bootstrapping technique. In this sample, the figure of the slope of best-fitting straight line between the variables presents a positive or negative correlation. The black line is the regression line without bootstrap, and consists of the predicted score on y for each possible value of x. The green lines show the range of change in the intercept and slope in resampling data during 1000 bootstraps. As is represented in Figure 5, the values of positive correlation between protein-T2M (0.4), protein-THI (0.37) show the positive slopes of the regression lines between these variables in Figure 7, but with a considerable scatter. The squared value of the correlation (0.16) allows for a quantification of linear dependency between predictand and predictor, explaining 14% of the total variability *vs.* remaining scatter, with 86% of total variability. For protein concentration *vs.* ETI, it changed to a negative correlation (-0.43), which is clear in Figure 7, as well leading to 18% for the linear dependency *vs*. 82% for unexplained scatter. In all cases, bootstrap increased the significance level of the best-fitted regression line. A question for future research is whether the amount of scatter might be significantly reduced by additional linear, or even nonlinear, predictors.

## 4. Conclusions

As mentioned in the introduction, one of the main objectives of this research is to evaluate the Pearson correlation under a statistical bootstrapping method for comparing the influence of dairy variable time series on climate time series.

The bootstrapped linear relationships between climatic parameters and milk compounds illustrate an interesting correlation between milk compounds and the climate parameters.

Although a nine year period data for milk compounds is not short dataset in agricultural investigations but in climate research field such a period is relatively short term. This methodology can be very useful for developing countries with short-term data sets. The bootstrapping technique is confirmed to the bivariate correlation in this study, which also agreed with results reported by Lunneborg [41]. The bootstrap results expanded confidence intervals while it demonstrates against the presented results of Rasmussen [42], which explained that bootstrapping overly restricted confidence intervals.

Additionally, when the distribution of available data sets is complicated or unknown, and only a small pilot sample with a great deal of uncertainty is available, one of the best solutions to estimate the regression coefficients and confidence interval of the regression equation is the bootstrap method. Applying the bootstrap technique is also very useful and relatively simple for analyzing what data collection is expensive, difficult, or data with gaps. Fundamentally, bootstrapping can be used for finding the regression coefficients in linear or non-linear models with an increasing significance level of the test.

With reference to the study of Silva and Maia [31], the Holstein breed in different climate conditions, such as tropical, Mediterranean, arid and semi-arid, has changed its characteristics*.* According to the characteristic changes in cows under different conditions, the effect of climate variability on animal products, new thermal stress indices with more significant probability and interaction, between animal physiology and climate variability, are needed.

The authors strongly suggest that more research in this field is needed in order to focus on discovering the critical points of climate indices regarding each of the milk components separately. Additionally, new indices have to consider more predictors, such as solar ration and percentage of fat and protein in the diet of cows, heat tolerance, how many days did cows suffer from extremely critical heat stress days, how much of the skin surface is covered with large black spots (especially for the Holstein breed), and also the ability of herd stations to control heat stress, facility of welfare, and breed of cows. Finally, every additional predictor can change the results and help to achieve models that are closer to reality.

In addition, to increase the usability of this technique, the authors propose that it makes sense to do further simulations in other climate regions, even given the statistical problems when using short time datasets with large amount of uncertainty.

Applying statistical modeling techniques would be an advantage in industry for the improvement of the food security and preventing expensive losses due to unexpected natural issues.
